# Liposomal Delivery Systems for Allergen-Specific Immunotherapy: From Molecular Design to Clinical Application

**DOI:** 10.3390/membranes16080277

**Published:** 2026-08-19

**Authors:** Daria N. Melnikova, Andrey E. Potapov, Daria S. Zavoiko, Tatiana V. Ovchinnikova

**Affiliations:** 1 M.M. Shemyakin & Yu. A. Ovchinnikov Institute of Bioorganic Chemistry, The Russian Academy of Sciences, 117997 Moscow, Russia; cool.goyan@yandex.ru (A.E.P.); daryazavoiko@yandex.ru (D.S.Z.); ovch@ibch.ru (T.V.O.); 2 Moscow Center for Advanced Studies, 123592 Moscow, Russia

**Keywords:** liposomes, allergen-specific immunotherapy, adjuvants, dendritic cells, regulatory T-cells, coiled-coil, CARPA

## Abstract

Liposomal systems are commonly used in drug delivery due to their low toxicity, biocompatibility and biodegradability. In this review, the influence of physicochemical parameters of liposomes, namely size, surface charge, and lipid composition, on biodistribution, dendritic cell uptake, and the character of the induced immune response is examined. The potential of strategies such as targeting C-type lectin receptors, the combined use of toll-like receptor agonists and tolerogenic molecules, and an approach based on high-affinity antigen binding to liposomes via coiled coil-forming peptides is evaluated. Mechanisms of tolerance induction at the humoral, cytokine, and cellular levels are discussed, including the switch from a Th2 to a regulatory T-cell response and the formation of blocking antibodies. Special attention is paid to safety aspects associated with the use of liposomal systems, specifically avoidance of pseudoallergic reactions linked to the complement system activation and the toxicity of cationic lipids, as well as approaches for minimization of these risks. The main obstacles to clinical application and promising directions of further research necessary for the development of effective and safe liposomal allergy vaccines are outlined. This review summarizes current data on the use of liposomal systems for allergen-specific immunotherapy.

## 1. Introduction

According to the World Health Organization (WHO) and the World Allergy Organization (WAO) reports, allergic diseases have become a global public health problem, reaching the scale of a non-infectious pandemic [1]. To date, allergen-specific immunotherapy (AIT) remains the only etiological treatment capable of altering the natural course of allergy and maintaining long-term tolerance [2,3].

AIT involves the repeated administration of escalating doses of an allergen adsorbed onto an adjuvant, which may include aluminum hydroxide, calcium phosphate or other mineral salts, microcrystalline tyrosine or other biodegradable amino acids. Despite its advantages, this approach has several limitations that restrict its broader use. Treatment courses last 3–5 years, and the risk of systemic anaphylactic reactions persists. Moreover, commonly used adjuvants promote polarization of the immune response toward a Th2 phenotype with a transient increase in IgE levels, which is undesirable for controlling allergic inflammation [2,3]. Consequently, active research is ongoing to identify alternative adjuvants and delivery systems for AIT. Ideal candidates must combine a high immunogenicity with a low allergenicity and enable a targeted modulation of the immune response toward a Th1 or regulatory T-cell profile.

Due to the possibility of tailoring their composition and size, liposomes can meet these requirements and are considered a promising platform for next-generation vaccines. Liposomes are non-toxic, biocompatible, and biodegradable. They can encapsulate hydrophilic molecules within their aqueous cores or adsorb them on their surfaces. In addition, they protect antigens from proteolytic degradation, which is particularly important for mucosal administration [4,5]. One key difference between liposomes and the widely used aluminum hydroxide is the targeted modification of the type of immune response. Instead of enhancing the Th2 response, liposomes can induce a Th1 or regulatory T-cell response, which is clinically preferable for suppressing allergies [6,7]. Encapsulation of the allergen physically shields its IgE-binding epitopes. This reduces the risk of mast cell and basophil degranulation and, consequently, the severity of anaphylaxis [8].

The aim of this review is to systematize current data on the use of liposomes in AIT. Key aspects determining the efficacy and safety of these systems are examined from their physicochemical properties and molecular mechanisms of interaction with the immune system to prospects for clinical application.

## 2. Advantages of Liposomes as a Platform for Allergen Delivery

More than 50 years ago, the ability of liposomes to enhance antibody production was first discovered. It was shown that negatively charged liposomes loaded with diphtheria toxoid induce significantly higher antibody titres than an equivalent dose of free antigen [9]. This study became the foundation for research into liposomes as allergen carriers to improve the efficacy and safety of AIT.

It was further demonstrated that repeated subcutaneous injections of the mugwort (*Artemisia scoparia*) pollen allergen Art v 1, encapsulated in multilamellar negatively charged liposomes, reduce the production of specific IgE in mice by nearly an order of magnitude and increase IgG levels compared to the free allergen [6]. Allergenicity of the formulation decreased by approximately ten-fold while immunogenicity was retained. Pharmacokinetic studies revealed prolonged retention of the encapsulated allergen in tissues, indicating the formation of a depot and prolonged release of the antigen [6]. Subsequent experiments showed that negatively charged vesicles capture the maximum amount of protein. Repeated injections of allergen encapsulated in liposomes result in lower IgE and higher IgG than injections of free allergen [10]. Additionally, mice immunized with liposome-encapsulated allergen survived repeated challenge, whereas animals receiving free allergen or allergen adsorbed onto aluminum hydroxide succumbed, likely due to systemic anaphylaxis. Plasma histamine levels in mice receiving the liposome-encapsulated allergen were significantly lower [11].

The main advantage of liposomes over conventional adjuvants is the ability to specifically modulate the polarization of the immune response. Aluminum hydroxide promotes Th2 cell differentiation and enhances IgE production, which is undesirable in the context of AIT. Liposomes can shift the balance toward a Th1 or regulatory T-cell response [6,7]. This is achieved both through the intrinsic properties of the lipid bilayer and through the ability to co-deliver immunomodulators. As part of a single particle, they enter the same antigen-presenting cells, providing a synergistic effect.

A second important benefit is physical shielding of IgE-binding epitopes. In a house dust mite allergy model, encapsulation of major allergens Der s 1 and Der s 2 in liposomes produced specific IgG levels comparable to those induced by aluminum hydroxide, while IgE levels were reduced by half and pulmonary eosinophilic infiltration was less pronounced [12]. Similar results were obtained for cationic liposomes conjugated with the birch allergen Bet v 1. Such carriers induced an earlier and stronger humoral response compared to aluminum hydroxide, as well as significant IL-10 production. Conjugation of Bet v 1 to the liposome surface reduced allergenicity approximately 15-fold relative to the free protein in the rat basophilic leukemia cell line degranulation assay [7].

Liposomes offer additional advantages. Their lipid composition can mimic cell membranes, resulting in high biocompatibility. Surface modification with ligands for dendritic-cell receptors enables active targeting [13,14]. The method of associating the allergen with liposomes, such as surface adsorption, encapsulation within the aqueous core, or a combination of both, affects particle stability, release kinetics, and the resultant immune profile. It has been shown that the combination of encapsulation and adsorption of Bet v 1 in cationic liposomes results in a stronger IgG1 response and increased production of IL-4, IL-5, IL-10, and IL-13 compared to either method used alone [8]. However, the realization of these advantages depends on the physicochemical characteristics of the liposomes.

These characteristics are largely determined by the preparation method. Common laboratory-scale techniques include thin-film hydration [15] followed by extrusion through polycarbonate membranes of defined pore size [16], which yields homogeneous populations of unilamellar vesicles with controlled diameters. Sonication is typically employed to produce small unilamellar vesicles [17], whereas repeated freeze–thaw cycles can increase encapsulation efficiency and lipid recovery [18]. Each method imparts distinct characteristics in terms of size distribution, lamellarity, and antigen loading capacity [17]. Therefore, the preparation protocol must be carefully selected and optimised for the specific allergen and the desired immunological outcome, as even minor variations in vesicle structure can significantly affect biodistribution, cellular uptake, and the resulting immune response.

## 3. Physicochemical Properties of Liposomes

The functionality of liposomes as adjuvants and carriers for AIT is determined by a combination of physicochemical parameters. The main ones include size, polydispersity, surface charge, lipid composition, mechanical rigidity, lamellarity, encapsulation efficiency, and release profile. Evaluation of these parameters is essential for quality control, batch-to-batch reproducibility, and the predictable efficacy of liposomal formulations *in vitro* and *in vivo*.

**Figure 1:**
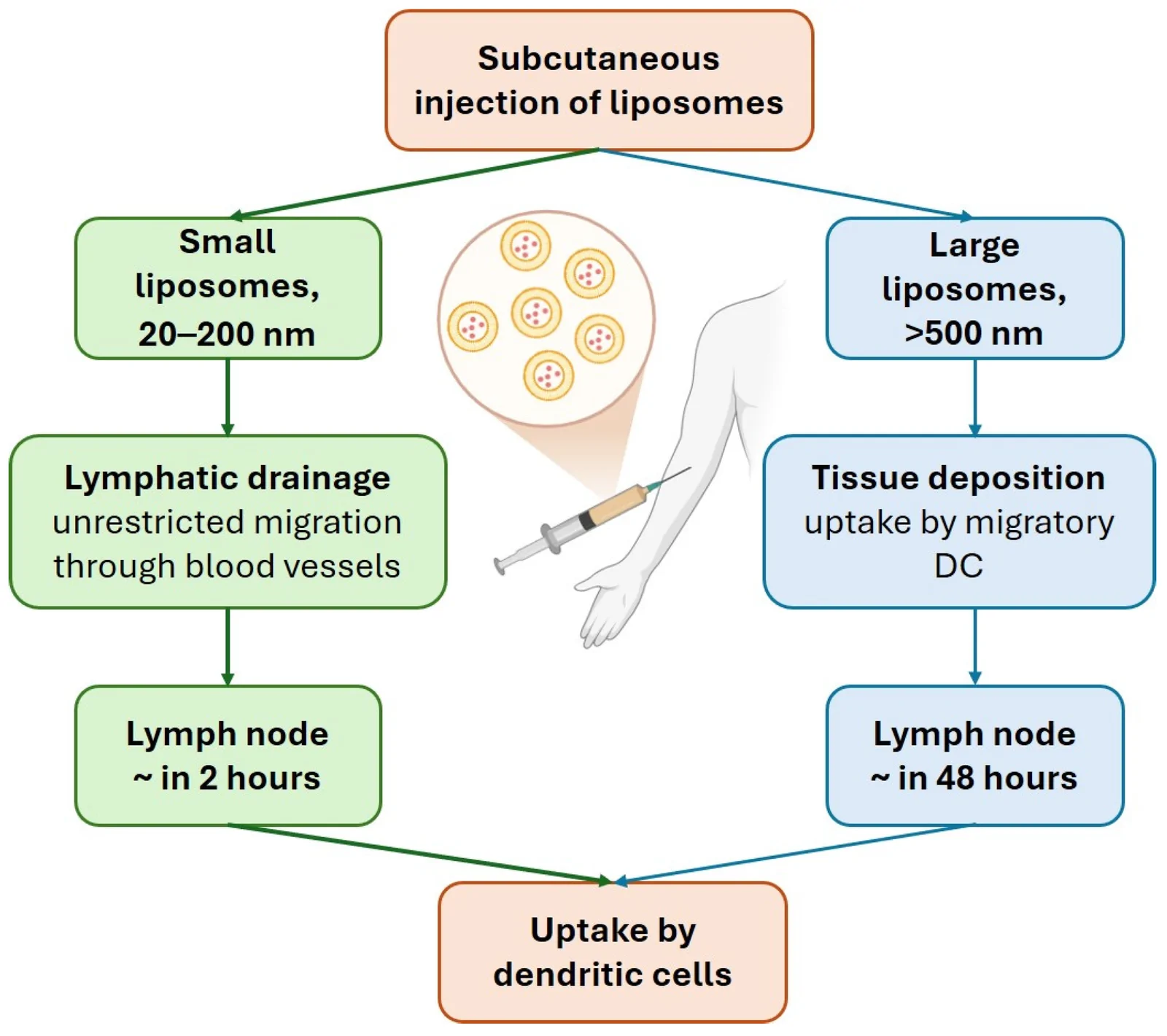
Schematic illustration of liposome uptake by dendritic cells as a function of size.

Liposome size influences their biodistribution and the mechanism of uptake by dendritic cells (Figure 1). Particles with a diameter of 20–200 nm, following subcutaneous administration, migrate through the lymphatic vessels and reach regional lymph nodes within two hours, where they are taken up by dendritic cells. Particles larger than 500 nm are retained at the injection site and transported by migrating dendritic cells, reaching the nodes only after 48 h [19,20]. The intermediate size range of 200–500 nm has also shown promise for dendritic cell targeting, with studies demonstrating a correlation between size, cellular uptake, and immune activity. Furthermore, the specific size within this range can determine the endocytic route and the intracellular processing of the antigen.

In most AIT studies, liposomes with diameters of 100–200 nm are used, as this range provides a balance between stability, effective uptake, and timely delivery to lymphoid organs [13,21]. However, size alone can influence the polarization of the immune response, at least in some models. Brewer et al. [21] demonstrated that vesicles ≥225 nm preferentially induced a Th1-biased response (IgG2a, IFN-γ) via macrophage IL-12 production, whereas vesicles ≤155 nm shifted the response toward a Th2 profile (IgG1, IL-5), with the transition occurring within 155–225 nm. When interpreted in the context of lymphatic drainage kinetics, the 100–200 nm range combines rapid lymphatic delivery to tolerogenic lymph node niches with the avoidance of the Th2 bias observed with smaller vesicles. Any residual Th2 tendency can be counterbalanced by the intrinsic Th1- or Treg-polarizing properties of anionic lipids (e.g., phosphatidylglycerol) [22] or by co-delivery of immunomodulators such as CpG-ODN [23] or vitamin D_3_ [24]. Nevertheless, these findings from Brewer et al. were obtained using ovalbumin-loaded liposomes in a murine model and may not be directly extrapolated to all liposome-allergen combinations, as the outcome also depends on lipid composition, charge, and other parameters.

The polydispersity index (PDI) serves as the standard quantitative measure of size distribution, where a PDI of 0.3 or lower is generally considered indicative of a homogeneous and pharmaceutically acceptable population. Conversely, samples with a high PDI are known to exhibit the variable cellular uptake and inconsistent therapeutic responses mentioned above [14].

Glycerophospholipids are the main components of the lipid bilayer. These can include both natural phosphatidylcholines and synthetic lipids (distearoylphosphatidylcholine (DSPC), dipalmitoylphosphatidylcholine (DPPC), dioleoylphosphatidylcholine (DOPC), and distearoylphosphatidylglycerol (DSPG)). The inclusion of cholesterol increases the packing density of the bilayer, enhances mechanical rigidity, and prevents nonspecific interactions with plasma proteins *in vivo* [4]. The lipid composition directly determines the adjuvant properties. Replacing the cationic lipid dimethyldodecylammonium (DDA) with the neutral DSPC weakens the Th1 response. Increasing the proportion of DSPC in DDA-TDB liposomes contributes to an undesirable shift toward Th2 [25]. Anionic liposomes are of particular interest for AIT because they can induce immunological tolerance. The efficiency of antigen-specific regulatory T-cell induction depends on the headgroup structure of the anionic phospholipid. Phosphatidylglycerol-based liposomes significantly outperform phosphatidylserine-based liposomes in inducing an activation of suppressive action of regulatory T-cells, despite having the same size and zeta potential [22].

The surface charge, characterized by the zeta potential, determines the electrostatic interactions between vesicles and their colloidal stability. Cationic liposomes (e.g., containing dioleoyl-3-trimethylammoniumpropane (DOTAP)) actively interact with the negatively charged cell membrane. They facilitate uptake by dendritic cells, promote their maturation, and enhance the production of pro-inflammatory cytokines [26]. However, there is no universal advantage to a cationic charge. When the allergen molecule is positively charged, the loading efficiency is higher for anionic liposomes [10,22]. In sublingual allergen immunotherapy, PEGylated liposomes based on neutral palmitoyl-oleoylphosphatidylcholine (POPC) or cationic lipid (e.g., DOTAP) with an encapsulated allergen were equally effective in suppressing eosinophilic airway inflammation. This indicates that the act of encapsulation itself plays a key role, rather than the charge [27]. Furthermore, in an ex vivo human skin model, anionic liposomes were taken up by dermal dendritic cells more efficiently than cationic ones, which calls into question the assumption that a cationic charge is advantageous for intradermal delivery [8]. Thus, the optimal charge depends on the route of administration and the physicochemical properties of the specific allergen.

In addition to surface charge, the mechanical rigidity of liposomes influences cellular uptake and the subsequent immune response. Atomic force microscopy has shown that the addition of cholesterol increases the rigidity of DOPC-based liquid-phase liposomes but decreases the rigidity of DSPC-based gel-phase liposomes. A direct correlation has been established between vesicle rigidity, their uptake by dendritic cells, and their ability to induce antigen-specific regulatory T cells *in vivo*: stiffer liposomes elicit a more pronounced Treg response. The exception is excessively soft liposomes, which lose the bound antigen before contact with the cells [28].

The structural features of vesicles also influence their functional properties. Lamellarity determines the encapsulation efficiency and the release kinetics. Multilamellar vesicles are characterized by longer retention of hydrophilic compounds compared to unilamellar vesicles. High encapsulation efficiency and loading capacity reduce allergen loss and allow a therapeutic dose to be achieved with a single administration. Controlled prolonged release of the allergen is necessary to maintain the tolerogenic signal and reduce the frequency of injections. For liposomes with covalently bound antigen, the determining factor is not the diffusion rate but the stability of the bond under physiological conditions [8].

The *in vivo* pharmacokinetics of liposomes are determined not only by their structural characteristics but also by their surface properties. In particular, PEGylation typically prolongs the circulation of vesicles in the bloodstream, but repeated injections can induce the formation of anti-PEG antibodies, predominantly IgM, and the associated phenomenon of accelerated clearance [27,29]. Large and cationic liposomes are more likely to activate the complement system, which manifests as pseudoallergic reactions (CARPA) [30,31]. These effects should be considered for AIT, as the treatment scheme involves multiple injections. Adverse reactions can be reduced by using anionic or neutral liposomes, limiting the PEG content to 5 mol. % or replacing it with alternative hydrophilic polymers, such as polyglycerol [5,27,31].

Thus, appropriate selection of size, charge, lipid composition, and mechanical rigidity enables the development of effective and safe platforms for AIT.

## 4. Current Advances in Liposomal Formulations for AIT

Based on the identified physicochemical principles, several approaches have been developed to enhance the immunogenicity of liposomal formulations and their ability to induce immunological tolerance. Dendritic cells play a central role in these strategies, as they are the main regulators of the immune response. As detailed below, they can either activate the immune system or induce tolerogenic responses depending on the specific stimuli. Targeted allergens administration to the dendritic cells can be used for decreasing the therapeutic dose and the risk of nonspecific additional effects.

The first generation of liposomal allergen formulations, based on multilamellar vesicles, was evaluated in several clinical studies. Multilamellar liposomes incorporating five-grass pollen or *Dermatophagoides pteronyssinus* extracts demonstrated significantly better cutaneous tolerance than aqueous extracts [32]. Empty liposomes were generally well tolerated locally and systemically, although delayed hypersensitivity reactions in two subjects were attributed to α-tocopherol used as an antioxidant [32]. Subcutaneous administration of encapsulated extracts resulted in 72% immediate local and 16% systemic reactions, a safety profile comparable to conventional immunotherapy [32]. A subsequent trial comparing liposomal five-grass pollen extract with calcium phosphate-adsorbed extract showed slightly superior clinical efficacy of the liposomal formulation but less favourable systemic safety [32]. These early findings, together with manufacturing complexity, led the researchers to conclude that liposomal allergen was not suitable at that time [32]. Nevertheless, these pioneering studies established proof-of-concept and identified key challenges such as local tolerability, systemic reactivity, and production scalability, that subsequent formulations have sought to address. Concurrently, Audera et al. confirmed the immunoadjuvant effect of liposomes for allergy immunotherapy and called for further pharmacokinetic and clinical studies [33].

These early studies highlighted the need for more sophisticated strategies to improve efficacy and safety. A major advancement was the development of targeted delivery systems designed to direct allergens specifically to dendritic cells, the central regulators of immune tolerance. One of the most extensively studied targeted delivery approaches is the modification of liposomes with ligands for C-type lectin receptors. The efficiency of this approach has been demonstrated in several experimental models. In particular, liposomes carrying the *Dermatophagoides farinae* allergen Der f 2 and conjugated to the oligosaccharide Lewis X were efficiently taken up by dendritic cells, resulting in suppression of IL-4, IL-6, and TNF-α secretion and a reduction in basophil activation [13]. In parallel, intradermal delivery of antigens using glycan-modified liposomes targeting the DC-SIGN receptor enhanced the antigen-presenting capacity of human CD14^+^ dermal dendritic cells; the addition of Lewis X further increased liposome uptake and CD8^+^ T-cell activation [34]. A specific glycomimetic ligand for langerin was also developed for selective targeting of human skin Langerhans cells, enabling efficient liposome delivery to these cells [35].

Another promising target is the DEC-205 receptor. Immunoliposomes conjugated with anti-DEC-205 antibodies exhibit enhanced uptake by monocytic dendritic cells more effectively than non-targeted analogues [36,37]. Efficient targeting can also be achieved through mannose receptors, as mannosylated liposomes actively interact with dendritic cells. Incorporation of the triterpenoid celastrol into such liposomes inhibits dendritic cell maturation by reducing CD80, CD86, and MHC-II expression, thereby diminishing their capacity to activate T cells; this produced a therapeutic effect in an experimental psoriasis model [38]. Although psoriasis is not an IgE-mediated disease, this approach demonstrates the feasibility of suppressing dendritic cell activation and reprogramming them into a tolerogenic phenotype using targeted liposomes. In the context of AIT, this strategy could be used to deliver allergens together with immunomodulators, thereby not only suppressing dendritic cell maturation but also promoting regulatory T-cell induction and the establishment of tolerance.

In addition to targeted delivery, modulation of innate immune signaling pathways represents another important strategy. Thus, allergen co-delivery with Toll-like receptor (TLR) agonists is an effective approach, which promotes a shift in the immune response from a Th2 to a Th1/Treg profile. Well-known TLR agonists include CpG oligodeoxynucleotides (CpG-ODN)—synthetic single-stranded DNA fragments containing unmethylated CpG dinucleotides. They selectively activate TLR9 because of structural similarity to bacterial DNA. In a murine asthma model, three subcutaneous injections of a liposomal formulation containing ovalbumin and CpG-ODN administered to animals with established allergic pulmonary inflammation led to the resolution of inflammation, conferred long-term protection, and reduced levels of specific IgE and IgG1. Neither the allergen alone nor CpG-ODN alone produced this effect. The protective action was completely dependent on the signaling adaptor MyD88 in CD11c-positive dendritic cells, as demonstrated in mice with selective deletion of MyD88 specifically in these cells [23]. The resolution of inflammation was not accompanied by an increase in the number of Foxp3^+^ regulatory T cells or IL-10-producing Tr1 cells in the lungs but required MyD88 signaling in dendritic cells. These data indicate a key role for dendritic cells in mediating the therapeutic effect [23].

In addition to TLR activation, the tolerogenic dendritic cell formation is necessary for the induction of sustained tolerance. Recent studies have demonstrated that liposomes loaded with vitamin D_3_ induce a tolerogenic phenotype in human monocyte-derived dendritic cells. This is characterized by a decrease in the CD86 and HLA-DR expression and an increase in the co-inhibitory immunoglobulin-like transcript 3 and programmed cell death ligand 1 expression. These dendritic cells have been shown to lead to the naïve CD4^+^ T-cells differentiation into Foxp3^+^ CD127^−^ Tregs, which express the TIGIT marker, thereby suppressing the Th1 and Th17 development [24]. Intradermally administered retinoic acid or vitamin D3-loaded liposomes ex vivo in human skin selectively induced the migration of CD14^+^ dermal dendritic cells expressing tolerogenic markers. These cells, in turn, promote the differentiation of functional Tregs [39]. Furthermore, it has been demonstrated that liposomes containing retinoic acid may also promote the differentiation of mucosal CD103^+^ dendritic cells. The suppression of the Th17-response is accompanied by the induction of IL-10-producing and various Foxp3^+^ Treg subpopulations, including ICOS^+^ Treg [40].

A passive mechanism associated with the use of liposomes enriched with phosphatidylserine (PS) has also been shown to make a significant contribution to the formation of the tolerogenic dendritic cell phenotype. Typically, PS is localized to the inner leaflet of the plasma membrane and is exposed on its outer surface only during apoptosis, where this molecule initiates phagocytosis and concurrently inhibits the development of an inflammatory response. PS on the liposomal surface may be used to mimic apoptotic cells. Thus, upon interaction with receptors on antigen-presenting cells (APC), these liposomes stimulate the TGF-β and IL-10 secretion that in turn promotes the Foxp3^+^ regulatory T-cells generation. It has been demonstrated that such liposomes, when loaded with ovalbumin, effectively suppress allergic airway inflammation and reduce specific IgE levels via different routes of administration [41,42].

To date, hybrid nanocarrier platforms combining liposomes with virus-like particles (VLPs), polymeric nanoparticles such as poly(lactic-co-glycolic acid) (PLGA), or exosomes have also been developed. A comparison of liposomes with these alternative platforms based on key AIT-relevant parameters is presented in Table 1. Hybrid platforms containing VLPs enable the design of highly immunogenic vaccines with low allergenicity that avoid anaphylactic responses [43,44]. The incorporation of PLGA particles into hybrids has been shown to facilitate a more regulated release of the allergen and adjuvant [45]. Previous studies have demonstrated that sublingual administration of mesenchymal stem cell exosomes loaded with ovalbumin effectively modulate the immune response and prevent the sensitization of the immune system in a mouse model of ovalbumin-induced allergic airway inflammation [46]. VLPs are lipid vesicles containing embedded viral proteins that only partially improved pulmonary mechanics parameters in an experimental asthma model. They also did not lead to complete resolution of the inflammatory process. This indicates the need for further optimization of these constructs [47].

A coiled-coil peptide-based platform represents a promising strategy for achieving controlled association of an antigen with the liposomal surface. In this system, a pair of heterodimeric peptides (pepE and pepK) is used: one peptide is covalently linked to a lipid anchor, most commonly cholesterol, and incorporated into the lipid bilayer of cationic liposomes, whereas the other is recombinantly fused to an antigen, such as ovalbumin or Bet v 1 [8]. The formation of an α-helical coiled-coil ensures antigen high-affinity binding to the carrier: the dissociation constant of the complex is 100–400 nM, which is much lower than that achieved with conventional electrostatic adsorption (>10^5^ nM). The approach yields a loading efficiency above 95% and imparts serum and *in vivo* stability. Immunization of mice with coiled-coil-based constructs has been shown to markedly enhance the CD4^+^ T-cell response: the EC_50_ for the T-cell proliferation *in vitro* was reduced 18-fold compared to the free peptide, and a significant increase in the proportion of CD4^+^ T-lymphocytes co-producing IFN-γ and IL-10, which is characteristic of a tolerogenic Th1 profile, was observed *in vivo* [8]. The combination of low allergenicity in basophil degranulation assays and high immunogenicity distinguishes this platform from conventional adjuvant systems and makes it one of the most clinically promising.

Broadening the range of cell targets has led to strategies not limited to dendritic cells. Targeting B lymphocytes responsible for allergen-specific IgE production is a promising therapeutic approach. The STALs (Siglec-engaging Tolerance-inducing Antigenic Liposomes) platform employs liposomes that co-display an allergen and a high-affinity ligand for the inhibitory receptor CD22 on their surface. Forced CD22 co-ligation with the B-cell receptor on allergen-specific B cells initiates signaling mediated by immunoreceptor tyrosine-based inhibitory motifs (ITIMs) in the cytoplasmic domain of CD22, which leads to B-cell apoptosis or anergy, clonal deletion, and IgE synthesis suppression. It has been demonstrated that the additional encapsulation of rapamycin into such liposomes substantially enhances the tolerogenic effect even in naïve animals [49,50].

Another approach directly targets the effector cells involved in allergic reactions. In that case liposomes co-display an allergen and a Siglec-8 receptor ligand on their surface and recruit this inhibitory receptor to the IgE–FcεRI complex on the surface of mast cells and basophils, thereby completely blocking degranulation. This treatment has been shown not only to prevent acute anaphylaxis, but also to induce prolonged desensitization of these cells to repeated allergen challenge [51]. Liposomes loaded with α-galactosylceramide (α-GalCer) are being actively investigated as a tool for shifting the cytokine balance. Presentation of α-GalCer to invariant natural killer T (NKT) cells via CD1d triggers robust IFN-γ production, thereby promoting the Th2 responses suppression and the attenuation of eosinophilic inflammation. In mouse models of asthma and allergic rhinitis, intranasal or sublingual administration of α-GalCer-containing liposomes led to an improvement in disease symptoms [52,53].

Lipid nanoparticles for mRNA delivery (mRNA-LNPs) represent another promising approach. Unlike recombinant proteins, mRNA-LNPs enable allergen synthesis directly at the site of administration, thereby reducing the likelihood of cross-linking with pre-existing IgE fixed to mast-cell surfaces and, consequently, decreasing the risk of anaphylaxis. Lipid nanoparticles targeted delivery to tolerogenic antigen-presenting cells is accompanied by a pronounced increase in Foxp3^+^ regulatory T cells. Suppression of allergic inflammation by this approach has been demonstrated in a mouse model of asthma [54], and its capacity to induce blocking IgG antibodies and ameliorate clinical manifestations has been shown in mouse models of food and pollen allergy [55].

In addition to active immunization with allergens, passive immunotherapy with monoclonal antibodies, for instance, omalizumab, which neutralizes free IgE, is used clinically for severe allergy. However, this approach requires repeated administration, fails to induce long-term tolerance, and does not target allergen-specific T cells. An alternative strategy is the induction of endogenous blocking antibodies using liposomal carriers. In this case, liposomes serve as carriers for chimeric peptide constructs containing B-cell epitopes from the constant domain of IgE, predominantly located in the Cε3 region responsible for FcεRI binding, or epitopes from the extracellular portion of FcεRIα, covalently linked to a carrier T-helper epitope, often derived from tetanus toxin. Immunization by liposomal carriers with chimeric peptides elicits the production of polyclonal IgG antibodies that bind free IgE and prevent its fixation on effector cells. The epitopes are selected to avoid cross-recognition of IgE already associated with FcεRI on the mast cells and basophils surface, thereby minimizing the risk of anaphylaxis. Long-term protection is achieved after a limited number of injections [56,57].

The comparison of the described liposomal platforms according to key characteristics is presented in Table 2.

Thus, regardless of the strategy chosen, the achievement of a clinical effect in AIT relies on the ability to induce a coordinated reprogramming of both the innate and adaptive arms of the immune system.

## 5. Immunomodulation Mechanisms in Liposomal Allergen Vaccines

**Figure 2:**
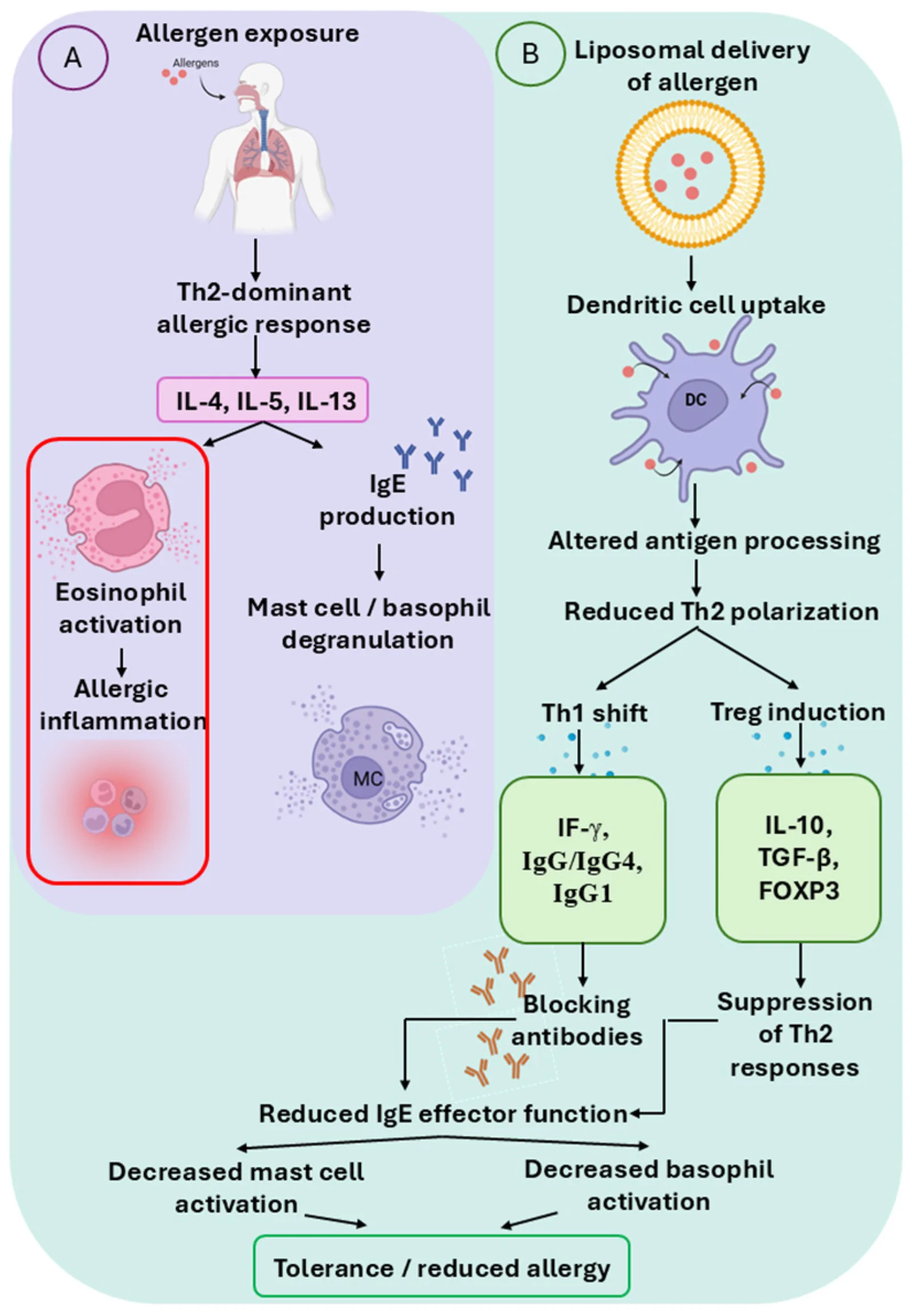
Schematic representation of immune deviation induced by allergen-specific immunotherapy. (**A**) In the absence of liposomal carriers, allergen exposure drives a Th2-polarised response, characterised by IgE production, eosinophilia, and Th2 cytokines such as IL-4, IL-5, and IL-13. (**B**) Liposomal allergen formulations redirect the response toward Th1 and regulatory T-cell (Treg)-mediated tolerance, promoting blocking antibodies (IgG, IgA), IL-10, IFN-γ, and FoxP3^+^ Tregs, while suppressing Th2 cells and eosinophils.

The efficacy of liposomal AIT is determined not only by the carrier design but also by the immunological shifts it elicits. It has been shown that AIT induces a complex remodeling of the adaptive and innate immune systems. This process includes at the molecular level increased blocking activity of IgG and IgA antibodies, long-term reductions in specific IgE levels and Th2 cytokine production (IL-4, IL-5, and IL-13), and augmented synthesis of IFN-γ and regulatory IL-10 (Figure 2A). At the cellular level, it encompasses the induction of regulatory T and B lymphocytes, the reprogramming of dendritic cells and innate lymphoid cells toward a tolerogenic state, and the suppression of effector cells such as mast cells, basophils, and eosinophils [58,59]. Liposomal allergen formulations can modulate all of these levels, and each of them is sensitive to the physicochemical properties of the carrier, including size, lamellarity, surface charge, and the presence of targeting ligands (Figure 2B).

The clinical efficacy of AIT is associated with increased blocking activity of IgG and IgA. Two major mechanisms mediate this effect. The first, receptor blockade, is based on the ability of IgG antibodies to engage Fcγ receptor IIb (FcγRIIb) on effector cells in proximity to FcεRI. In this way, an allergen simultaneously bound by IgG and IgE on FcεRI induces co-ligation of FcεRI and FcγRIIb, resulting in ITIM-mediated inhibition of cellular activation. The second mechanism, steric blockade, involves a competition between IgG, IgA, and IgE for binding to allergens [58,59]. Particularly, in humans, IgG4, is considered the major blocking antibody subclasses, and their levels increase markedly during successful AIT [60,61]. Liposomes enhance this process through several mechanisms. The controlled allergen release, achieved by selecting the lipid composition and lamellarity, provides prolonged antigenic stimulation, promoting the accumulation of IgG blocking subtypes. For instance, liposomal formulations based on coiled-coil associations have been observed to induce an early and potent increase in IgG1 and IgG2a that surpasses the response to allergens associated with aluminum hydroxide [7]. In sublingual AIT, α-galactosylceramide-containing liposomes increased not only IgG2a but also mucosal IgA, whereas IgE and IgG1 levels decreased [53]. A number of liposomal formulations, notably those containing α-GalCer, have been shown to attenuate the transient escalation of specific IgE that is characteristic of the initial phase of conventional AIT. This phenomenon can be attributed to rapid allergen internalization by antigen-presenting cells and reduced allergen availability to free IgE [53].

It has been shown that changes in the humoral profile during AIT are accompanied by shifts in the cytokine balance. IL-10 plays a pivotal role in the development of tolerance; it is secreted by CD4^+^ T lymphocytes, regulatory B cells, and type 2 innate lymphoid cells (ILC2s). In experiments with liposomal allergy vaccines, IL-10 levels have been observed to increase consistently in the lymph nodes, spleen, and bronchoalveolar lavage fluid [62,63,64]. The dynamics of IFN-γ, a key Th1 cytokine, remain poorly defined. The available evidence is inconclusive: some studies report an increase [53,62], whereas others find no significant change [23]. These inconsistencies can probably be explained by variations in both immunization protocols and the lipid composition of the nanocarriers. Cationic liposomes incorporating CpG-ODN, for example, trigger a robust IFN-γ response, while neutral vesicles that contain phosphatidylserine act through IL-10-mediated mechanisms. Concurrently, the suppression of Th2 cytokines (IL-4, IL-5, and IL-13) has been observed in virtually all models and is among the most reproducible effects. The IL-10-to-IL-4 ratio has been identified as a promising integrated marker of efficacy, particularly for the liposomal formulations. In comparison to aluminum hydroxide-based adjuvants, liposomal formulations exhibit a substantially higher IL-10-to-IL-4 ratio.

The cytokine profile is a direct manifestation of the profound changes occurring at the cellular level. The induction of Foxp3^+^ Tregs and IL-10-producing Tr1 cells has generally been regarded as the key AIT mechanism. As demonstrated in a study [64], liposomes loaded with ovalbumin induced CpG islands demethylation in the *FOXP3* promoter (up to 55%). This effect was more pronounced with oligomannose-modified liposomes that facilitated delivery to the dendritic cell mannose receptors. Eventually, epigenetic reprogramming has been associated with the Treg-mediated tolerance. However, Tregs might play no major role in the symptom improvement and immunological changes at the early timepoint of the treatment or in these systems. It is therefore hypothesized that regulatory B-cells and IL-10^+^ ILC2s may exert the dominant suppressive function during the early phase [65]. Thus, a more long-term course of treatment lasting approximately three years will result in a significant increase in Foxp3^+^ Tregs.

Liposomes targeting CD206 and TLR2 have been shown to polarize bone marrow-derived dendritic cells into a semi-mature tolerogenic phenotype (CD86^low^MHC II^high^). These cells have been observed to secrete IL-10 and TGF-β, thereby creating an anti-allergic environment within the lymph nodes [64]. AIT has been demonstrated to induce a shift in ILC2 toward protective IL-10^+^KLRG1^+^ phenotype and to inhibit proallergic seasonal increase in IL-13^+^ ILC2s [66,67]. While the direct impact of liposomes on this process remains to be elucidated, the capacity of liposomal formulation to enhance IL-10 and TGF-β local concentrations establishes the conditions for immune response changes. Administration of liposomal allergen formulations has repeatedly been shown to reduce the number of eosinophils in the mucosa and bronchoalveolar lavage fluid that correlates with a decrease in IL-5 levels [23,53,62]. The observed effect may be linked to two potential factors: firstly, a reduction in the allergen availability, and secondly, the direct suppressive lipid compounds activity.

Consequently, liposomal allergen vaccines induce a multifaceted immune response encompassing antibody production, cytokine modulation, and cellular regulation. This response is characterized by the lipid carrier physicochemical properties. In parallel, Th2 cell activity and pro-inflammatory mediator production decrease, while the response shifts toward a tolerogenic phenotype that suppresses allergen-specific immunity. Collectively, these changes result in sustained tolerance development to the allergen.

## 6. Safety of Liposomal Allergen Delivery Systems

Liposomes possess potent immunomodulatory activity, making safety considerations highly relevant. Importantly, allergen encapsulation reduces the risk of IgE-mediated anaphylaxis, the dominant safety concern in AIT. In support of this, encapsulated ovalbumin elicited a significantly weaker reaction than free ovalbumin in a mouse model of active cutaneous anaphylaxis. A liposomal formulation containing the allergen and CpG-ODN has been shown to confer long-term protection against allergic lung inflammation. Similar results were obtained with the *Blomia tropicalis* extract [23]. In experiments involving escalating doses of liposomal allergen, mice survived, whereas animals receiving either free allergen or allergen adsorbed to aluminum hydroxide did not. This effect may be associated with markedly lower plasma histamine levels following liposomal administration [11].

In addition to IgE-mediated reactions, liposomes themselves and their components may induce non-IgE-mediated hypersensitivity. A clinically important consequence in this regard is complement activation-related pseudoallergy (CARPA), which is triggered by complement activation. In the bloodstream, liposomes can activate the complement cascade, leading to the anaphylatoxins C3a and C5a release. These anaphylatoxins are thought to act as the key mast cell degranulation inducers, however, other cell populations may contribute to the developing reaction. Collectively, these events lead to systemic hemodynamic alterations that are clinically indistinguishable from anaphylaxis yet occur in the absence of prior IgE sensitization [30]. The risk of developing CARPA increases with the use of cationic liposomes and large particles. In the case of PEGylation, although it does not directly activate complement and may reduce complement interaction during the first administration, repeated administration can induce the formation of IgM antibodies to PEG, which then trigger complement activation and accelerate drug elimination, thereby increasing the risk of developing CARPA during multiple injections typical of AIT [34].

These findings are in line with earlier reports in the KU812 human basophil model, demonstrating that large and positively charged vesicles more readily trigger cell activation [68]. Thus, the main difficulty is to reconcile the adjuvant liposome action with the pseudoallergy risk. A rational strategy may be the use of anionic or neutral liposomes ranging from 100 to 200 nm, containing either low levels of PEG or alternative hydrophilic polymers.

It should be noted, however, that most published data on CARPA and the ABC phenomenon derive from intravenous administration studies, where liposomes enter the systemic circulation directly and rapidly [30,31]. In AIT, subcutaneous and mucosal (sublingual or intranasal) routes are preferred. Subcutaneous administration leads to gradual absorption via the lymphatic system, with significant particle retention at the injection site or in draining lymph nodes, whereas mucosal routes expose only a fraction of the dose to the systemic circulation. Consequently, the risk of systemic CARPA is expected to be lower for these extravascular routes than for intravenous injection. Nevertheless, the ABC phenomenon has been observed not only after intravenous but also after subcutaneous injection of PEGylated formulations [69], indicating that anti-PEG IgM production can occur independently of the administration route. Therefore, while the absolute risk of systemic CARPA is reduced for subcutaneous and mucosal administration, the potential for local complement activation at the injection site or in mucosal tissues, as well as the induction of anti-PEG antibodies following repeated dosing, cannot be completely dismissed, particularly given the multi-injection schedules typical of AIT [27,29].

In addition to pseudoallergic reactions, the potential toxicity of lipid components should also be considered. Natural phospholipids, such as phosphatidylcholines, are biocompatible and are metabolized through endogenous pathways. Cationic lipids are often required for the efficient loading of negatively charged nucleic acids or allergens and may exhibit concentration-dependent cytotoxicity, particularly towards phagocytic cells [70,71]. For clinical application, it is advisable to employ biodegradable cationic lipids analogues and to strictly control the administered dose.

Thus, the development of safe liposomal systems for AIT requires a balance between efficacy and potential adverse reactions.

## 7. Conclusions

To date, liposomal allergen formulations have been shown to represent a promising strategy for allergen-specific immunotherapy. Their principal advantage over classical adjuvants lies in the ability both to reduce allergenicity by shielding IgE epitopes and to redirect the immune response from a pathogenic Th2 profile toward a regulatory or Th1-mediated one. At the same time, the final outcome is determined not by a single parameter but by the combination of physicochemical properties of the carrier, each of which exerts a distinct influence on the immunological response.

Current liposome engineering approaches, including targeting of dendritic cells, co-delivery of immunomodulators, use of tolerogenic molecules, and high-affinity antigen anchoring to the liposomal surface, expand the possibilities for controlling membrane properties and improve therapeutic efficacy in preclinical models. However, most of these data have been obtained in prophylactic settings, whereas the clinically relevant scenario involves treatment of already sensitized patients. Accordingly, translation of findings from preventive models to therapeutic ones is not always straightforward.

Safety remains a central issue in the development of liposomal formulations. On the one hand, antigen encapsulation substantially reduces the risk of anaphylaxis, as demonstrated experimentally. On the other hand, liposomes, particularly cationic and PEGylated formulations, may trigger pseudoallergic reactions through the complement system activation. These risks can be reduced by rational optimization of formulation compositions, but no universal solution is currently available, since optimal parameters depend strongly on the specific allergen and the route of administration.

Clinical experience with liposomal allergovaccines remains limited due to a small number of studies, and to date, no product has been approved for broad clinical use. This indicates that clinical application is feasible, but a widespread implementation is hindered by the absence of standardized quality-control approaches, the lack of validated biomarkers, and an insufficient understanding of long-term effects. Particular attention probably has to be paid to mucosal routes of administration, as they may be safer in the context of repeated dosing.

Thus, liposomes possess a substantial therapeutic potential. However, its realization will require a transition from preclinical studies to clinical application of well-characterized formulations with a demonstrated efficacy in therapeutic models and in rigorously designed randomized trials. Further progress in this field will depend not only on the development of new carrier types but also on a deeper understanding of mechanisms underlying allergen tolerance, identification of predictive markers of treatment response, and establishment of unified standards for evaluating such products at all stages of development. A key point of these investigations has to be focused on the systematic examination of an influence of the physical properties of the lipid membrane on the immune response development. This will help to translate liposomal systems from preclinical development into practical clinical application.

## Figures and Tables

**Table 1:** Comparative characterization of liposomes and other nanocarriers for AIT.

Parameter	Liposomes	PLGA-Particles	VLPs	Exosomes
**Material**	Phospholipids (natural or synthetic) + cholesterol [4,5]	PLGA—synthetic biodegradable copolymer [45]	Viral structural proteins (e.g., capsid proteins) lacking genetic material [43,44]	Endogenous membrane vesicles secreted by various cells [46]
**The size**	50–300 nm (for AIT more frequently 100–200 nm) [13,21]	100–400 nm (controlled during the synthetic procedure)	20–200 nm (depends on the donor virus) [43,44]	50–200 nm (natural, depends on the donor cell) [46]
**Biocompatibility**	High (lipids are the cell membrane components)	High (biodegradable polymer, US Food and Drug Administration (FDA)—approved for several applications)	Moderate (viral proteins can be immunogenic upon repeated administration)	High (endogenous origin, low immunogenic)
**Antigen release control**	Moderate (depends on lamellarity, lipid composition, and rigidity; release over hours–days) [8]	High (tuned by the monomer’s ratio and molecular weight; release over days to weeks) [45]	Complicated (antigen is typically displayed on the surface or encapsulated within the capsid; release is difficult to control)	Natural (the release profile is donor-cell-dependent and difficult to standardize)
**Immunogenicity**	Modulatable (can direct the immune response towards Th1, Treg, or Th2 by altering charge, size, and lipid composition) [6,7]	Moderate (frequently requires an adjuvant to elicit strong immunity)	High (provides strong B- and T-cell responses but retains a risk of allergenicity) [43,44]	Variable (depends on the donor cell; generally tolerogenic, but immunogenic under certain conditions) [46]
**CARPA risk**	Moderate (elevated for cationic and large particles >200 nm) [30,31]	Not studied (no systematic data available)	Not studied (no systematic data available)	Not studied (no systematic data available)
**Active targeting capability**	High (surface can be modified by antibodies, glycans, peptides and lipids) [13,38]	Moderate (requires ligands chemical conjugation; maintaining activity is problematic)	High (surface proteins can be genetically or chemically modified)	High (can be engineered via donor cell modification or post-isolation conjugation)
**Production scalability**	High (the production methods scale easily)	High (the production methods scale easily)	Moderate (the production methods depend on cell culture, thereby restricting scalability and overall yields)	Low (the production methods scale poorly, yielding low output and high batch-to-batch variability)
**Stability *in vivo***	Moderate (rapid clearance; PEGylation prolongs circulation but may elicit an immune response)	High (slow release, but particles can accumulate in the liver and spleen)	Moderate (depends on the virus type, may elicit neutralizing antibody secretion)	Low (rapid clearance from the bloodstream, short circulation period)
**Clinical status for AIT**	Single random clinical trial (RCT) [48]; no approved drug	Preclinical studies (sensitized murine models) [45]	Preclinical studies (murine models) [43,44]	Preclinical studies (murine models) [46]

**Table 2:** Key characteristics of liposomal platforms for AIT.

Platform	Physicochemical Principle	Immune Effect	Key Advantage	Main Limitation
**Neutral liposomes**	Electrically neutral bilayer (POPC, DOPC, DSPC) with low reactivity toward plasma proteins	Balanced immune activation; IL-10-dependent suppression upon mucosal administration	Minimal toxicity; no complement activation	Weak antigen association; low cellular uptake
**Cationic liposomes**	Positively charged membrane (DOTAP, DDA) enabling electrostatic interactions with cells and antigens	Efficient dendritic cell uptake, strong humoral response (IgG1/IgG2a), reduced allergenicity	High antigen loading (>95% in the coiled-coil platform); Th1 skewing with CpG	Toxicity of cationic lipids; risk of CARPA after intravenous administration
**Anionic liposomes**	Negatively charged membrane (DSPG, phosphatidylglycerol) with receptor-mediated uptake	Induction of Foxp3^+^ Treg cells; nearly tenfold reduction in IgE	Stability, low cytotoxicity, tolerogenic effect	In some models, higher IgE than with neutral liposomes
**Targeted liposomes**	Surface ligands for C-type lectin receptors (DC-SIGN, DEC-205, mannose receptor, langerin)	Selective dendritic cell uptake, suppression of proinflammatory cytokines, reduced CD80/CD86/MHC-II expression	High specificity, reduced off-target delivery, potential dose reduction	More complex manufacturing; strong dependence on ligand density
**Liposomes with co-delivered adjuvants**	Antigen plus immunomodulator (CpG-ODN, vitamin D3, retinoic acid, phosphatidylserine, α-GalCer) within the same particle	CpG induces Th1 responses via TLR9/MyD88; vitamin D3, retinoic acid, and phosphatidylserine promote Treg responses; α-GalCer induces IFN-γ via NKT cells	Synergy through delivery to the same APCs; suppression of transient IgE increase	Risk of excessive activation; difficult control of particle composition

## Data Availability

No new data were created or analyzed in this study. Data sharing is not applicable to this article.

## Abbreviations

The following abbreviations are used in this manuscript:

AIT	allergen-specific immunotherapy
CARPA	complement activation-related pseudoallergy
CD	cluster of differentiation
CpG	cytosine-phosphate-guanine
CpG-ODN	CpG-oligo-deoxynucleotides
DC-SIGN	dendritic cell-specific intercellular adhesion molecule-3-grabbing non-integrin
DDA	dimethyldioctadecylammonium
DEC-205	dendritic and thymic epithelial cell receptor-205
DNA	deoxyribonucleic acid
DOPC	dioleoylphosphatidylcholine
DOTAP	dioleoyl-3-trimethylammoniumpropane
DPPC	dipalmitoylphosphatidylcholine
DSPC	distearoylphosphatidylcholine
DSPG	distearoylphosphatidylglycerol
EC_50_	half maximal effective concentration
EE	encapsulation efficiency
FcεRI	high-affinity IgE receptor
Foxp3	forkhead box P3
HLA-DR	human leukocyte antigen DR
ICOS	inducible co-stimulator
IgA	immunoglobulin A
IgE	immunoglobulin E
IgG	immunoglobulin G
IgM	immunoglobulin M
IL	interleukin
IFN-γ	interferon-γ
KLRG1	killer cell lectin-like receptor G1
LC	loading capacity
MHC	major histocompatibility complex
MLV	multilamellar vesicles
mRNA	messenger ribonucleic acid
MyD88	myeloid differentiation primary response protein 88
PEG	polyethylene glycol
TDB	trehalose 6,6′-dibehenate
TGF-β	transforming growth factor β
Th	T helper
TIGIT	T-cell immunoreceptor with immunoglobulin and immunoreceptor tyrosine-based inhibitory motif domains
TLR	Toll-like receptor
TNF-α	tumor necrosis factor α
Tr1	type 1 regulatory T cells
Treg	regulatory T-cells
